# Hybrid online-flipped learning pedagogy for teaching laboratory courses to mitigate the pandemic COVID-19 confinement and enable effective sustainable delivery: investigation of attaining course learning outcome

**DOI:** 10.1007/s43545-021-00117-6

**Published:** 2021-04-26

**Authors:** Ahmed M. Elkhatat, Shaheen A. Al-Muhtaseb

**Affiliations:** Department of Chemical Engineering, Qatar University, P.O. Box 2713, Doha, Qatar

**Keywords:** Pedagogy, Flipped learning, Lab courses, Online teaching, COVID-19

## Abstract

Since the early spring of 2020, the coronavirus pandemic (COVID-19) outbreak has hindered traditional face-to-face teaching and hands-on, traditional delivery of laboratory courses, forcing universities to migrate from the traditional way of teaching to a remote online approach. Although few studies addressed the pandemic's impact on educational outcomes, no studies are found to investigate the impact of the remote online teaching approach on laboratory courses. This paper highlights the impact of the online teaching approach, coupled with flipped learning pedagogy, as a substitute for traditional laboratories. The course learning outcomes and assessment tools are analyzed and discussed for 46 students enrolled in the Unit Operations Laboratory course in the chemical engineering program at Qatar University. Results show that the course learning outcomes are achieved effectively using the hybrid online-flipped learning pedagogy, which can be considered for computerized traditional laboratories as a moderation solution to alleviate pandemic COVID-19 confinement on learning outcome. This methodology can also be sustained in the future to facilitate the teaching of such lab courses, even in normal conditions, to optimize the resources and avail the delivery of such courses to a larger audience who may have various obstacles to attending traditional lab courses.

## Introduction

Teaching laboratories are a dynamic portion of engineering undergraduate programs, where most of the hands-on experiential learning occurs in the laboratory. The curricular objective of teaching laboratories is to relate theoretical principles and practice, offer students a visual sense of physical units, and help them develop the "feel for engineering" (Bisantz and Paquet 2002; Flack and Volino 1999; Johnson et al. 1995; Okamura et al. 2002; Olinger and Hermanson 2002; Leva 2003; Moore and Voltmer 2003). Teaching laboratories involve higher levels of learning activities, including the promotion of knowledge, comprehension, application, analysis, synthesis, and evaluation of facts from different perspectives according to Bloom's taxonomy (Armstrong; Darwazeh 2017). Hence, laboratory courses support profound cognition of fundamentals and theoretical principles through practical observations and investigations and support constructive learning strategies that help students absorb different subjects into their metacognition (Burnett et al. 2004; Eklund-Myrskog 1998; Lee et al. 2009; Lin and Tsai 2009; Marshall et al. 1999; Marton et al. 1993; Purdie and Hattie 2002; Roger 1979; Tsai 2004).

Since the early spring of 2020, the pandemic coronavirus COVID-19 outbreak has hindered global personal contacts for various purposes. Governments worldwide have taken precautionary measures, with emphases on social distancing and working from home, to hamper the virus spread and ensure citizens' safety. The education sector has been affected dramatically, and the responses of universities varied due to the pandemic. While some universities suspended the teaching until further notice or postponed the start of the summer semester (Impact of COVID-19 on studying abroad in Europe: Overview 2020; How is COVID-19 affecting schools in Europe? 2020), others have suspended all on-campus activities, including face-to-face teaching and replaced them by online and remote education.

The remote teaching of online lab courses provides many compensations. It provides a safe alternative for investigational operations that might have safety risk considerations, and it helps reduce the required asset and maintenance costs and retain the lab space (Baher 1999; Lee et al. 2002; Svajger and Valencic 2003). Besides, it helps students who are geographically dispersed and provides accessibility to students who have disabilities that may affect their potential to access and operate physical lab equipment. Despite the concerns about the quality of learning outcomes of online laboratories compared to conventional hands-on laboratories, most empirical studies declare that online laboratories' learning outcomes are as good as conventional ones, and students who were engaged in remote lab education gained conceptual knowledge satisfactorily (Gustavsson et al. 2009; Kostaras et al. 2011; Lindsay and Good 2005; Nedic et al. 2003; Nickerson et al. 2007; Sicker et al. 2005). Besides, conducting the experiments in a teamwork-environment provides a collaborative experience and disengages any potential isolation perceived from the online learning process (Hoyer et al. 2004; Sebastian et al. 2003).

The impact of the COVID-19 epidemic on teaching labs' educational outcomes is not clear yet. Although numerous publications exist in the literature examined the epidemic impact on psychological behavior among undergraduates (Dhar et al. 2020; Maqsood et al. 2021; Chaturvedi et al. 2021; Tang et al. 2020) and on medical education (Hung et al. 2020; Liu et al. 2020; Loch et al. 2021; Shih et al. 2020), no empirical study currently exists that evaluated the impact of the COVID‐19 on online Lab education, especially in engineering curricula. In this paper, we study how have hybrid online-flipped learning pedagogy of teaching laboratory courses impacted course learning outcomes in the Unit Operations Laboratory course in the chemical engineering program at Qatar University to alleviate the pandemic's impact COVID-19 confinement. The flipped teaching strategy, also known as blended, reverse, and inverted learning or classroom strategies (Bergmann and Sams 2012), is a relatively recent education methodology. It was known firstly as "Inverted classroom" (Lage et al. 2000; Steed 2012), where students prepare for the class by studying the material independently in advance, and then utilize the class time to further discuss the related skills and concepts (Garrison and Vaughan 2013; Hung 2014). This current study includes assessing Course Learning outcomes (CLOs) that are mapped to the Student Outcomes (SOs) of the Accreditation Board for Engineering and Technology (ABET) for 46 students enrolled in the Unit Operations Laboratory course, a part of the undergraduate program in Chemical Engineering at Qatar University using determined Assessment Tools (ATs). The study was conducted in Spring 2020 to perceive how hybrid online teaching coupled with a flipped learning approach affects the corresponding educational outcomes' attainment.

## Methodology

The online lab content was designed and prepared from scratch. The theoretical principles were taught on a whiteboard in the corresponding lab room to give students the same feeling of actual lab experience that they were used to before the pandemic. This was followed by explaining the experimental setup and then operating the equipment according to the prescribed experimental procedure. All these parts were filmed, wherein the length of each part of the video was designed to be within the recommended duration that should not exceed 20 min (Fadol et al. 2018). The videos were then edited professionally, uploaded on YouTube (Ahmed Mohamed Elkhatat 2020a, b), and the corresponding playlist link was announced to the students on the Qatar University's Blackboard platform (Alcorn et al.).

Online teaching was coupled with the flipped learning strategy to maximize the online teaching approach's benefits. Students were requested to watch the posted video in advance, while the assigned online lab time was devoted to developing students' critical thinking through collaborative discussions and in-depth problem-solving tutorials. Although leading collaborative discussions is much more challenging than delivering a traditional lecture, its effectiveness is higher than traditional lectures. (Kletz 2006). It is noteworthy that this teaching strategy might not succeed if students did not watch the video before attending the online lab session (Chen et al. 2014; Hao 2016; Lai and Hwang 2016). Hence, a pre-lab quiz was assigned at the beginning of each online session. Pre-lab quizzes were designed in multiple-choice question (MCQ) format to examine the students' understanding of the general principles covered in the video. The pre-lab quizzes utilize the Blackboard online testing tool, and students were given five minutes to finish the pre-lab quiz. At the end of the online lab session, each team receives a set of raw data collected from the experiment, and the team leader of each group was requested to assign the report tasks among his team members.

**Fig. 1:**
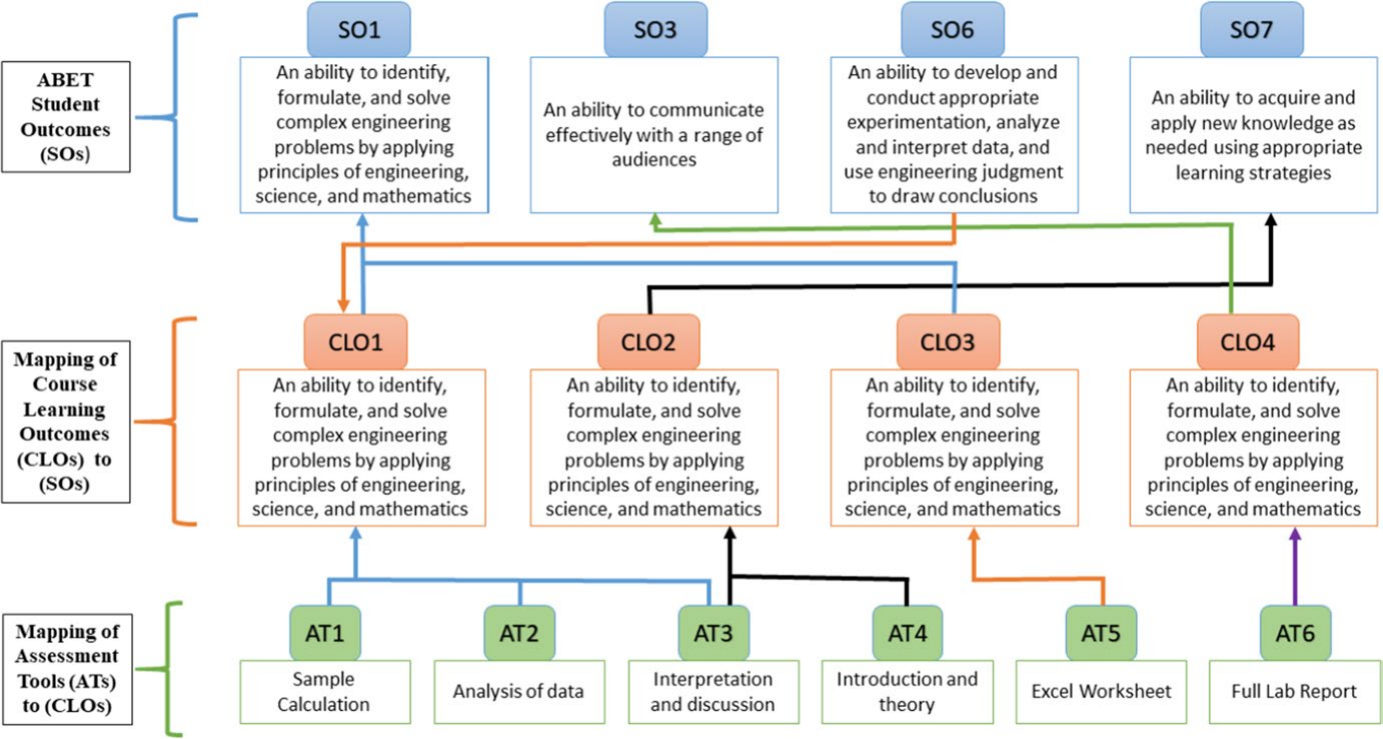
Holistic mapping of assessment tools (ATs) to course learning outcomes (CLOs) to ABET students learning outcomes (SOs)

The impact of the hybrid flipped-online lab course approach on the students' learning achievements was evaluated through six assessment tools (ATs) that measure four-course learning outcomes (CLOs) that are mapped to four student outcomes (SOs) of the (ABET) (ABET 2019). The four ABET student outcomes, SO1, SO3, SO6, and SO7, evaluate students' ability to solve complex engineering problems, communicate effectively with a range of audiences, develop and conduct appropriate experimentation, and acquire and apply new knowledge as needed, respectively. The course learning outcomes mapped to ABET student outcomes CLO1, CLO2, CLO3, and CLO4, evaluate student's ability to analyze experimental results by utilizing acquired technical engineering knowledge, utilize technical literature to obtain the required physical properties, use appropriate software to solve equations and interpret experimental results, and prepare professional technical reports, respectively. These outcomes were measured using six assessment tools; (AT1), which is the sample calculation section in the lab report; (AT2), which is the analysis of data section in the lab report; (AT3), which is the interpretation and discussion section in the lab report; (AT4), which is the introduction and theory section in the lab report; (AT5), which is the Excel worksheet that contains calculations, tables and charts; and (AT6), which is the overall appraisal of the lab report that includes (in addition to the previous sections) report presentation, spelling and grammar, abstract, experimental setup/procedure, raw data, conclusions, and citations and references. Figure 1 illustrates the holistic mapping of assessment tools (ATs) to course learning outcomes (CLOs)to ABET students learning outcomes (SOs).

It is noteworthy that two other CLOs for this lab course were ignored in this study. One of them measures students' ability to safely and effectively carry out experiments in a group setting, and the other one measures students' ability to design experiments to measure specific data. The first CLO was considered while using the traditional teaching approach in Labs, but the hybrid online-flipped approach is assumed to be safe. Besides, students are also preparing the lab report in the same group setting throughout the semester. Hence this CLO becomes inapplicable in this study. On the other hand, the other CLO was measured separately as a homework assignment, so it is practically independent of the teaching method.

In this work, an empirical investigation of course learning outcomes was studied utilizing a pool of 46 chemical engineering students enrolled in the Unit Operations Laboratory course. A quasi-experimental method was adopted, in which two teaching approaches were used in the same semester and for the same students. A traditional lab approach was used in teaching three experiments (EXP1 through EXP3) before the COVID-19 confinement, while a hybrid online-flipped lab approach was used in teaching four experiments (EXP4 through EXP7) during the COVID-19 confinement, as indicated in Table 1.

**Table 1:** Types of teaching approaches used for each experiment

Experiment	Experiment code	Teaching week	Teaching approach
Cooling tower	EXP1	19th–23th January 2020	Traditional
Tray dryer	EXP2	26th–30th January 2020	Traditional
Molecular diffusion in gases	EXP3	2nd–6th February 2020	Traditional
Wetted-wall gas absorption	EXP4	8th–12th March 2020	Hybrid (online-flipped)
Fixed and fluidized bed	EXP5	15th–19th March 2020	Hybrid (online-flipped)
Distillation column	EXP6	22th–26th March 2020	Hybrid (online-flipped)
Gas absorption	EXP7	29th March–2nd April 2020	Hybrid (online-flipped)

Two minor limitations associated with the current study should be considered with their contribution and can be alleviated in future research. The first limitation is that this study was conducted on female students only. Ideally, both genders should be involved in future studies to investigate any potential differences between their educational experiences and validate the anticipated equality in educational attainments. The second limitation is that the study should be reproduced in appropriate circumstances without potential physiological effects such as the predominance of stress and anxiety among home-quarantined students who are affected by the pandemic COVID-19 confinement, to validate the impact of the hybrid online-flipped teaching approach on the attainment of CLOs (Dhar et al. 2020; Maqsood et al. 2021; Chaturvedi et al. 2021; Tang et al. 2020).

All ATs, CLOs, and SOs data were standardized into a continuous scale of 0 to 100-point distribution and presented in Tables 2, 3, and 4. This distribution provides a more precise assessment and is more sensitive to differences among different ratings, making them easier to interpret (Cummins and Lau 2005; Nietfeld et al. 2006). The 100-point distribution was achieved by applying the following equation:

$${X}_{\text{S}}=\frac{X-{S}_{\text{min}}}{{S}_{\text{max}}-{S}_{\text{min}}}*100,$$

where ${X}_{\text{S}}$ is the standardized score, $X$ score to be converted, ${S}_{\text{min}}$ is the minimum score possible on the scale, ${S}_{\text{max}}$ is the maximum score possible on the scale.

**Table 2:** Analysis of students’ performance through assessment tools (ATs) of each experiment (at 80% IC)

Experiment code	AT1	AT2	AT3	AT4	AT5	AT6	AT average
EXP1	74.78 ± 5.97	72.39 ± 5.73	62.17 ± 5.95	84.35 ± 4.72	74.78 ± 5.97	71.16 ± 5.54	73.27 ± 5.65
EXP2	87.39 ± 4.6	82.61 ± 4.36	69.67 ± 7.57	85.65 ± 3.91	87.39 ± 4.6	71.35 ± 4.47	80.68 ± 4.92
EXP3	90.22 ± 4.5	95.65 ± 3.85	81.52 ± 4.95	93.48 ± 3.94	90.22 ± 4.5	86.95 ± 3.8	89.67 ± 4.26
EXP4	98.26 ± 1.54	89.35 ± 3.06	76.74 ± 4.29	89.57 ± 4.71	98.26 ± 1.54	86.01 ± 2.51	89.7 ± 2.94
EXP5	96.96 ± 1.36	93.04 ± 2.59	74.78 ± 6.1	100 ± 0	96.96 ± 1.36	87.28 ± 2.12	91.5 ± 2.26
EXP6	84.35 ± 3.14	92.61 ± 3.28	94.78 ± 1.66	94.35 ± 1.7	84.35 ± 3.14	91.71 ± 2.02	90.36 ± 2.49
EXP7	100 ± 0	93.91 ± 2.59	96.96 ± 1.36	100 ± 0	100 ± 0	94.83 ± 0.82	97.62 ± 0.8

**Table 3:** Analysis of students’ performance through course learning outcomes (CLO) of each experiment (at 80% IC)

Experiment code	CLO1	CLO2	CLO3	CLO4	CLO average
EXP1	69.78 ± 5.88	73.26 ± 5.34	74.78 ± 5.97	71.16 ± 5.54	72.25 ± 5.68
EXP2	79.89 ± 5.51	77.66 ± 5.74	87.39 ± 4.6	71.35 ± 4.47	79.07 ± 5.08
EXP3	89.13 ± 4.43	87.5 ± 4.44	90.22 ± 4.5	86.95 ± 3.8	88.45 ± 4.29
EXP4	88.12 ± 2.96	83.15 ± 4.5	98.26 ± 1.54	86.01 ± 2.51	88.88 ± 2.88
EXP5	88.26 ± 3.35	87.39 ± 3.05	96.96 ± 1.36	87.28 ± 2.12	89.97 ± 2.47
EXP6	90.58 ± 2.69	94.57 ± 1.68	84.35 ± 3.14	91.71 ± 2.02	90.3 ± 2.38
EXP7	96.96 ± 1.32	98.48 ± 0.68	100 ± 0	94.83 ± 0.82	97.57 ± 0.71

**Table 4:** Analysis of students’ performance through ABET student outcomes (SOs) of each experiment

Experiment code	SO (1)	SO (3)	SO (6)	SO (7)	SO average
EXP1	72.28 ± 5.93	71.16 ± 5.54	69.78 ± 5.88	73.26 ± 5.34	71.62 ± 5.67
EXP2	83.64 ± 5.06	71.35 ± 4.47	79.89 ± 5.51	77.66 ± 5.74	78.14 ± 5.2
EXP3	89.68 ± 4.47	86.95 ± 3.8	89.13 ± 4.43	87.5 ± 4.44	88.32 ± 4.29
EXP4	93.19 ± 2.25	86.01 ± 2.51	88.12 ± 2.96	83.15 ± 4.5	87.62 ± 3.06
EXP5	92.61 ± 2.36	87.28 ± 2.12	88.26 ± 3.35	87.39 ± 3.05	88.89 ± 2.72
EXP6	87.47 ± 2.92	91.71 ± 2.02	90.58 ± 2.69	94.57 ± 1.68	91.08 ± 2.33
EXP7	98.48 ± 0.66	94.83 ± 0.82	96.96 ± 1.32	98.48 ± 0.68	97.19 ± 0.87

This standardized score also represents the following traditional percentage grade cutoff scale of the course evaluation system in compliance with academic grading in the United States universities and Qatar University (Qatar_University 2020; NAEP 2011) and reflects the achievement components and level of performance at each assessment tool:

- ${X}_{\rm S}\ge 89.5\%$; represents an excellent performance and demonstrates independent thought and critical reflection on the related issues.
- ${89.5\%>X}_{\rm S}\ge 84.5\%$; represents a very good performance and demonstrates a considerable amount of critical thought and independence for the related issues.
- ${84.5\%>X}_{\rm S}\ge 79.5\%$; represents a good performance and demonstrates a concrete critical thought, analytical ability, and understanding of the related issues.
- ${79.5\% >X}_{\rm S}\ge 74.5\%$; represents a satisfactory performance and demonstrates a clear understanding of the topic and an ability to engage with the debates in the related issues critically.
- ${74.5\% >X}_{\rm S}\ge 69.5\%$; represents an adequate performance and demonstrates a sufficient understanding of the topic although average ability to engage with the debates in the related issues critically.
- ${69.5\% >X}_{\rm S}\ge 64.5\%$; represents a limited performance and demonstrates a fair understanding of the topic, although the average ability to critically engage with the debates of the related issues.
- ${64.5\% >X}_{\rm S}\ge 59.5\%$; represents a minimal performance, where minimum academic criteria are met. Besides, it demonstrates a minimum understanding of the topic with the lowest degree of judgment and independent thinking.
- ${59.5\% >X}_{\rm S}$: represents a poor performance and demonstrates an absence of both judgment and independent thinking.

A confidence interval estimate (CI) of 80% (Gardner and Altman 1986; Lee 2016; Walter 1995) was applied to interpret and appraise the empirical investigation for its validity and applicability. Cl is an interval within which the parameter is expected to fall with a certain degree of confidence. The general formula is

$${\text{Sample}}\;{\text{mean}} \pm \left( {{\text{Critical}}\;{\text{ value}}*{\text{Estimated}}\;{\text{standard}}\;{\text{error}}} \right)$$

The sample mean is the best point estimate, and it is the center of the confidence interval, and it is calculated as the arithmetic average of the data. The Critical value (*Z*-value) for 80% CI (*α* = 0.2) is 1.28. The sample size is the number of students involved in the study (46 students), and the estimated standard error is the average value of the error and was calculated according to the following formula:

$${\text{Estimated}}\;{\text{ standard}}\;{\text{error}} = \frac{{{\text{Standard}}\;{\text{deviation}}}}{{\sqrt {{\text{Sample}}\;{\text{of}}\;{\text{size}}} }}$$

For example, (from Table 3). The CLO1 of the EXP1 at 80% CI is 69.78 ± 5.88 means 80% of the data is located in the interval of 69.78 ± 5.88(i.e., between 63.9 and 75.66).

## Results and discussion

**Fig. 2:**
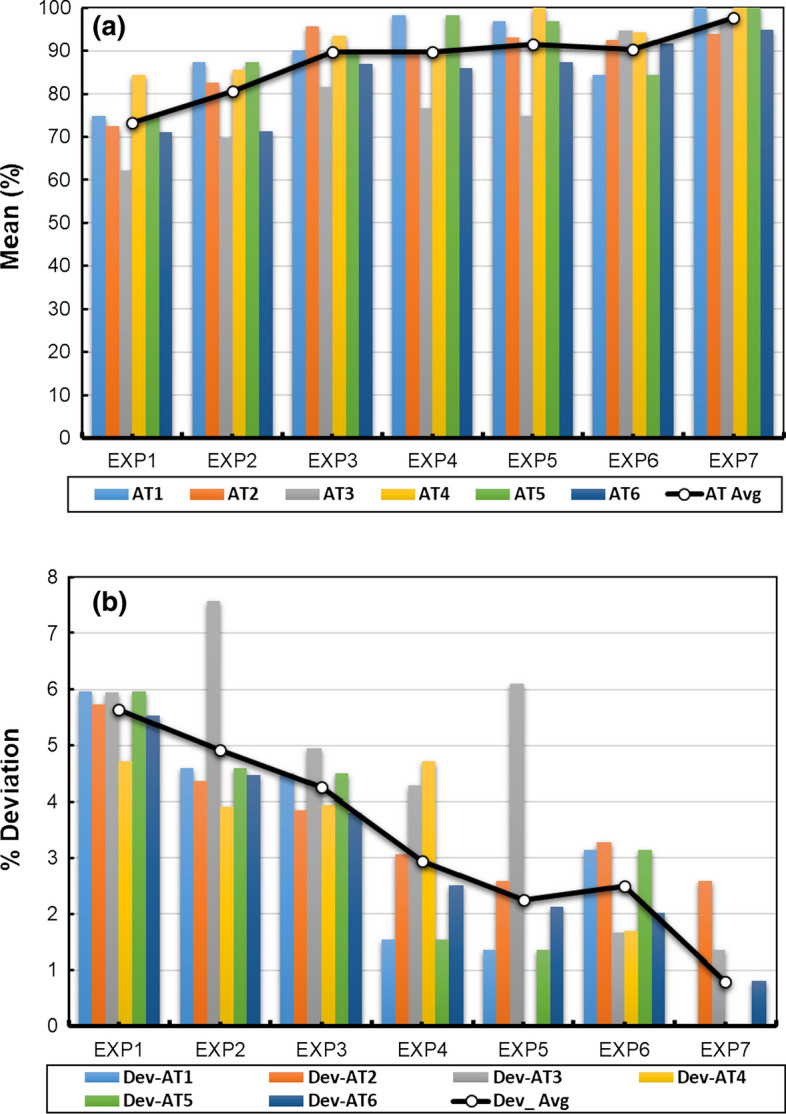
Appraising the students’ attainment in each experiment in terms of **a** AT means and **b** AT deviations within 80% CI

**Fig. 3:**
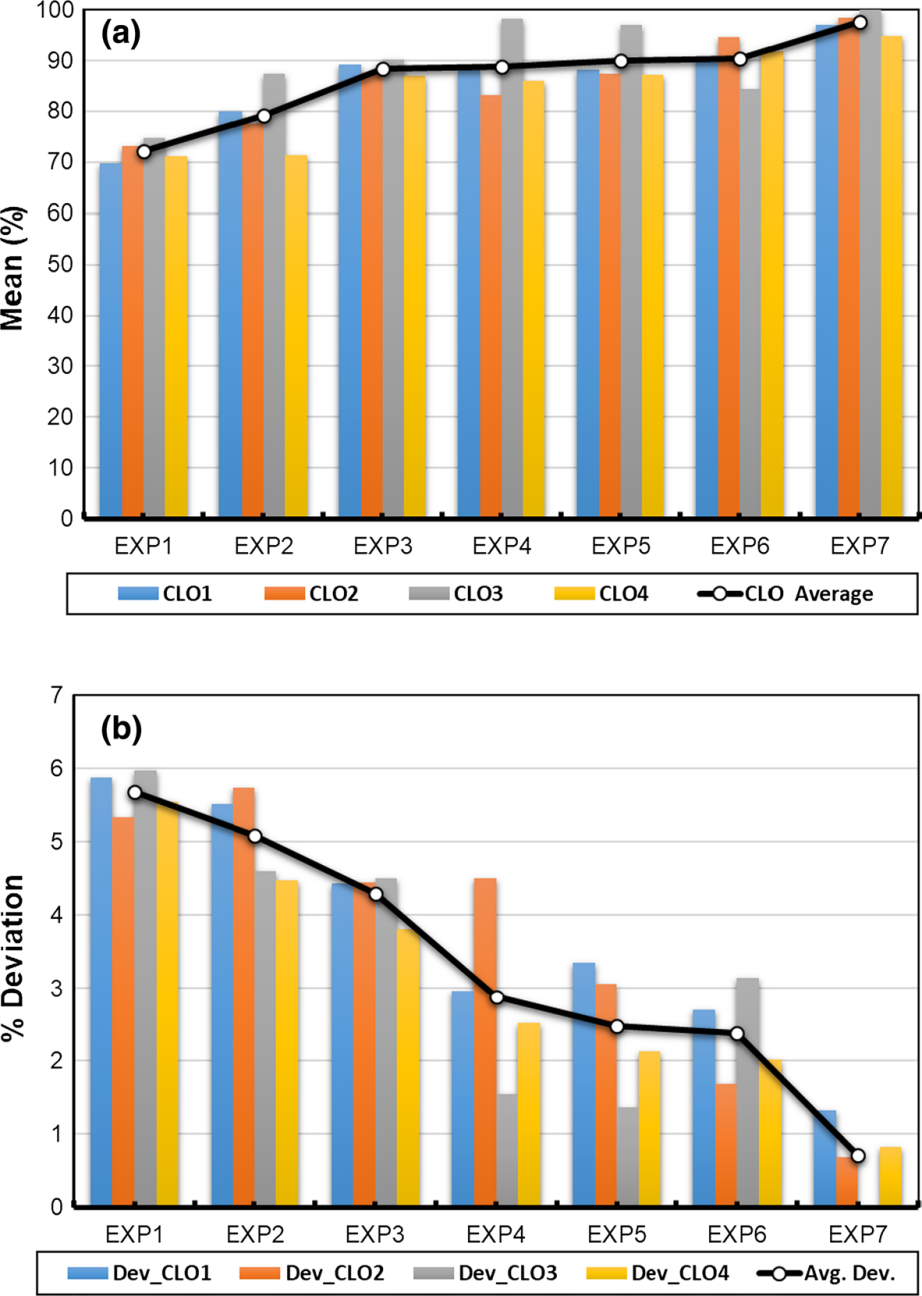
Appraising the students’ attainment in each experiment in terms of **a** CLO means and **b** CLO deviations within 80% CI

Curriculum Learning Outcomes (CLOs 1, 2, 3 and 4) of the hybrid online-flipped teaching approach are mapped to four ABET student outcomes (SOs 1, 3, 6, and 7) and were evaluated using identified assessment tools (ATs 1, 2, 3, 4, 5, and 6). Each assessment tool was assessed based on a checklist of detailed rubrics developed and announced to students (Ahmed M. Elkhatat 2020a, b), except (AT5), which was evaluated based on the calculation and figures performed in the submitted Excel worksheet. The students' overall performance in each assessment tool (AT) throughout the experiments (EXP1 through EXP7) is presented in Table 2 and Fig. 2. The Curriculum Learning Outcomes (CLO) throughout the experiments (EXP1 through EXP7) is presented in Table 3 and Fig. 3.

The interpretation and discussion section in the lab report represented by (AT3), which evaluates the Curriculum Learning Outcome (CLO1), the student's ability to analyze experimental results by utilizing acquired technical engineering knowledge, shows a slight fall in the first two weeks of online Lab teaching (EXP4 and EXP5) from 81.52 ± 4.95 to 76.74 ± 4.29 and 74.78 ± 6.1. This fall was expected due to the sudden switch in the teaching method from face-to-face and hands-on practice to online, especially since the interpretation and discussion section is considered the most demanding part of the report, as it needs deep and critical thinking, coupled with utilizing technical literature to interpolate and discuss the results (Masoud 2017). However, adopting the hybrid online-flipped teaching approach helped mitigate sudden migration unfamiliarity with online teaching platforms and shortened the recovery time to acquire the learning outcome. Giving students enough time to prepare for the experiment in advance utilizes the online lab session to discuss the related skills and concepts further. Besides, the students' teamwork, critical thinking, and lifelong learning skills were boosted through the collaborative discussions in the flipped teaching approach, impacting students' interpretation and discussion section and shortened the (AT3) delay for two weeks. The favorable impact of flipped teaching is apparent when the (AT3) of EXP6 (94.78 ± 1.66) and EXP7 (96.96 ± 1.36) are compared with (AT3) of EXP1 (62.17 ± 5.95), EXP2 (69.67 ± 7.57), and EXP3 (81.52 ± 4.95).

On the other hand, the other two assessment tools measure course learning outcome CLO1, AT1, and AT2, which correspond to sample calculation and data analysis sections in the lab report, respectively, were not affected by switching to the online-flipped approach. AT2 shows a slight drop from (95.65 ± 3.85) to (89.35 ± 3.06) in the first week of switching before recovering quickly in the subsequent weeks (93.04 ± 2.59 trough 93.91 ± 2.59). Conversely, AT1 did not show any fall but rose from (90.22 ± 4.5) to (98.26 ± 1.54), reflecting the positive impact of flipped teaching approach on students' ability to analyze experimental results. The hybrid online-flipped teaching approach helps students interpret each experiment's significant outputs and correlates them with theoretical principles supported with references that underpin their arguments professionally. The obtained results agree with other studies on the influence of flipped class on the learning outcome (Akçayır and Akçayır 2018; O'Flaherty and Phillips 2015; Pierce and Fox 2012; Gomez-Tejedor et al. 2020).

Moreover, the student's ability to utilize technical literature to obtain the required knowledge and physical properties represented by the course learning outcome (CLO2), evaluated based on assessment tools AT3 and AT4 that measure the interpretation and discussion section and the introduction and theory section, respectively, was affected partially by the switch in the teaching approach. Although the online-flipped teaching approach influence (AT3) as discussed earlier, it has no impact on (AT4). This is because the introduction and theory section requires students to review the literature on the experiment, and the overall improvement in (AT4) is because students became familiar with rephrasing techniques of rewording the introduction and theory section.

The hybrid online-flipped teaching approach exhibits a measurable effect on the course learning outcome (CLO3) that assesses students' ability to use appropriate software to solve equations and interpret experimental results. This CLO that was evaluated using the student's Excel Worksheet (AT5) shows improvement in students' performance from (90.22 ± 4.5) to (98.26 ± 1.54) on the first week of the new teaching approach. In addition to the hybrid online-flipped teaching approach, this growth is also coupled with students' awareness of using the Microsoft Excel software to solve equations, analyze data, and interpret experimental results.

The improvement in preparing professional technical reports reflected in (CLO4) and assessed by a full lab report (AT6) demonstrates continuous improvement throughout the course from (71.16 ± 5.54) to (94.83 ± 0.82) throughout the seven experiments. This improvement is due to the assorted efforts done by students' contributions in all report sections, supported with the hybrid online-flipped learning approach, which enables a sustainable performance of the lab experiments effectively and helps mitigate the pandemic COVID-19 confinement.

**Fig. 4:**
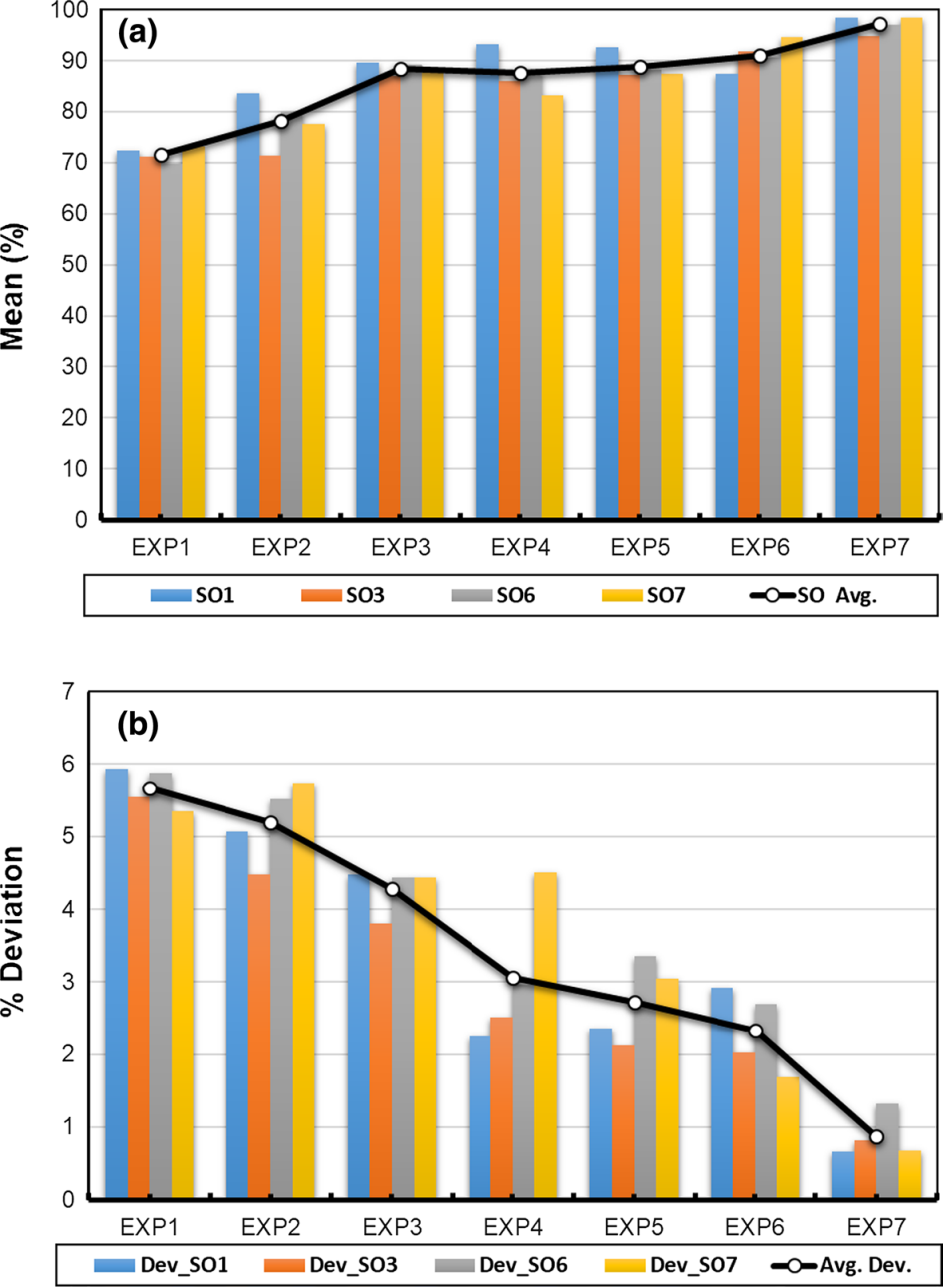
Appraising the students’ attainment in each experiment in terms of **a** SO means and **b** SO deviations within 80% CI

The other interesting finding in this study is that the online-flipped learning approach reduces the academic gap among students declined throughout the course progress, illustrated in Tables 2 and 3 and Figs. 2b and 3b. The averages of ClO and AT deviations within the 80% confidence interval have declined (from 5.68 to 0.71) and (from 5.65 to 0.8), respectively. Correspondingly, the overall students' performance, evaluated through ABET student outcomes (SOs), illustrated in Table 4 and Fig. 4, shows how the hybrid online-flipped teaching approach helps develop attaining the relevant SOs and reducing the academic gap among students throughout the experiments.

## Conclusion

The hybrid online-flipped teaching approach can be considered a potent approach to mitigate the destructive effect of stopping the traditional lab teaching approach on learning outcomes. Results show that the flipped teaching approach helped mitigate the sudden switch in the teaching method from face-to-face and hands-on practice to online, and it recovered the migration unfamiliarity with online teaching platforms. Besides, it boosted the students' teamwork, critical thinking, and learning skills through the collaborative discussions in the online sessions. Generally, the hybrid online-flipped lab teaching approach helped to achieve a continuous development of attaining the relevant course learning outcome throughout the experiments. Despite some temporary slight reductions in attaining some demanding learning outcomes within the first week of the switch in the teaching approach, particularly, the student's ability to analyze experimental results by utilizing acquired technical engineering knowledge, the student's ability to prepare professional technical reports, and the student's ability to utilize technical literature to obtain the required knowledge and physical properties, the flipped approach helped reduce the recovery time required to attain the learning outcome. Even it improved them in the subsequent weeks. Students' ability to appropriate software for solving equations and interpreting experimental results showed a continuous improvement without delay due to the switch in teaching mode. This is because of students' awareness of using the Microsoft Excel software. Furthermore, the academic gap among students decreased significantly after adopting the hybrid teaching approach. Although the overall improvement in the student's ability to utilize technical literature to obtain the required knowledge and physical properties, this improvement can be attributed to the assorted efforts done by students' contributions in all report sections, hence flipped approach has a minimum impact on this learning outcome. Despite this study's mentioned limitations, the hybrid online-flipped teaching approach is a promising lab pedagogy approach. It can be sustained in the future to facilitate the teaching of lab courses, even in normal conditions, to optimize resources and avail the delivery of such courses to a larger audience who may have various obstacles to attending traditional lab courses.

## Data Availability

All data analysed are contained in the paper.
